# Increased *Demodex* Density in Patients Hospitalized for Worsening Heart Failure

**DOI:** 10.3390/jpm10020039

**Published:** 2020-05-13

**Authors:** Serkan Yüksel, Esra Pancar Yüksel

**Affiliations:** 1 Cardiology Department, Faculty of Medicine, Ondokuz Mayıs University, 55139 Samsun, Turkey; 2 Dermatology Department, Faculty of Medicine, Ondokuz Mayıs University, 55139 Samsun, Turkey; esrapancar@yahoo.com

**Keywords:** *Demodex* mites, demodicidosis, heart failure, immunosuppression

## Abstract

Infection is an important factor leading to the exacerbation of heart failure (HF), resulting in hospitalization. *Demodex* species are obligatory parasites in human skin, and increased density was reported in immunocompromised patients. In this study, we aimed to investigate the *Demodex* density in hospitalized HF patients compared to that of healthy controls. Methods: This study included 36 HF patients and 36 age and sex-matched healthy controls. Five standardized biopsies were taken from the face of participants and assessed for *Demodex* by a light microscope. Results: At least one *Demodex* mite was detected in 20 HF patients and nine of the control group. The number of *Demodex* mites was significantly higher in the HF group (median 1; min. 0 and max. 10) compared to the control group (median 0; minimum. 0 and maximum. 3). Demodicidosis was positive in 14 of the HF patients. Demodicidosis was not detected in the control group. Conclusions: This study showed that *Demodex* positivity is more common in HF patients hospitalized for HF exacerbation. Demodicidosis should be considered in hospitalized HF patients.

## 1. Introduction

Heart failure (HF) is defined by the presence of typical symptoms and signs caused by structural and functional cardiac abnormality, resulting in reduced cardiac output and/or elevated intracardiac pressures [1]. The prevalence of HF in the adult population is approximately 1–2% [2,3,4,5]. In the elderly population, heart failure is one of the most common causes of hospital admissions [6,7]. Infections are one of the leading precipitating factors that are responsible for exacerbation in HF patients. They have been shown to be associated with increased short-term mortality in HF patients compared to other precipitating factors, such as atrial fibrillation and hypertension [8,9,10].

*Demodex folliculorum* and *Demodex brevis* are obligatory parasites and usually found in the hair follicles and pilosebaceous glands of human skin [11,12]. They were commonly detected on the facial skin especially, forehead, cheeks, nose, and nasolabial fold [12,13]. Their incidence was reported to be as high as 93% [11]. *Demodex* mites could be detected in healthy individuals, but the density is low. *Demodex* mites are accepted as pathogenic when their number exceeds the five mites/cm^2^ of skin [14,15]. Their growth might be facilitated by some local or systemic factors [16,17]. In the literature, increased *Demodex* densities were reported in immunocompromised patients with leukemia and acquired immune deficiency syndrome and also those under immunosuppressive treatments [17,18,19]. Besides the immunocompromised patients, increased *Demodex* density was also reported in patients with end-stage renal failure [20,21].

In this study, we aimed to investigate the *Demodex* density in hospitalized patients with HF exacerbation and compared those with age and sex-matched healthy controls.

## 2. Materials and Methods

### 2.1. Study Population

This study was conducted between January and March 2020. Among seventy-seven HF patients who were admitted to emergency and outpatient clinics for worsening heart failure, forty-one patients with malignancies, end-stage renal failure, facial erythematous lesions, whose ages were older than 75 years because of the high rate of reported *Demodex* positivity [22], and who were unable to consent or unwilling to participate were excluded from the study. The final study population included 36 HF patients (25 male, 11 female; mean age 67 ± 7 years) hospitalized for the exacerbation of HF and the control group consisted of age and sex-matched 36 healthy individuals (22 male, 14 female; mean age 64 ± 6 years). The institutional ethics committee approved the study protocol with code number OMU-KAEK-2020-32 on 16.01.2020. All participants provided written informed consent. This study was conducted in accordance with Declaration of Helsinki. 

### 2.2. Demodex Investigation

Standardized skin surface biopsies (SSSBs) were taken from the forehead, nose, chin and, cheeks of all participants. For biopsy procedure, a drop of cyanoacrylate glue was put on a 1-cm^2^ marked area of a slide. The glue-bearing side of the slide is applied over the skin for 30 s. Then, the slide was gently removed from the skin, 2–3 drops of immersion oil were applied. The slides were investigated for parasites under a light microscope at ×10 and ×40 magnifications. When at least one *Demodex* mite was detected, the test was accepted as positive. Demodicidosis was considered when five or more parasites in a 1-cm^2^ area were detected [14].

### 2.3. Statistical Analysis

The continuous variables with normal distribution were presented as mean ± standard deviation values, those without normal distribution as median (minimum and maximum). The categorical variables were presented as percentages. For the analysis of the normal distribution of the variables, the Kolmogorov-Smirnov and Shapiro–Wilk tests were used. The comparison of continuous variables with normal distribution, the student *t*-test was used. The Mann–Whitney U test was used to compare those without normal distribution. The Chi-square test and Fisher’s exact test were performed for comparison of categorical data. All statistical analyses were performed using the SPSS version 20 (SPSS Inc, Chicago, IL, USA). All statistical tests were two-sided, and a *p*-value < 0.05 was accepted as statistically significant.

## 3. Results

A total of 36 patients with HF and age, sex-matched 36 healthy controls were included in this study. The etiology of HF was ischemic heart disease in 17 (47%) patients. The mean left ventricular ejection fraction (EF) of HF patients was 40% ± 14%. The types of HF were HFREF (Heart Failure with Reduced Ejection Fraction) in 19 (53%) and HFPEF (Heart Failure with Preserved Ejection Fraction) in 17 (47%) patients. The reasons for HF exacerbation were fluid retention due to noncompliance with medications and diet in 19 (53%) patients, infections most commonly pneumonia in seven (19%) and arrhythmias in six (17%) patients. The clinical and laboratory parameters of HF patients were presented in Table 1.

**Figure 1:**
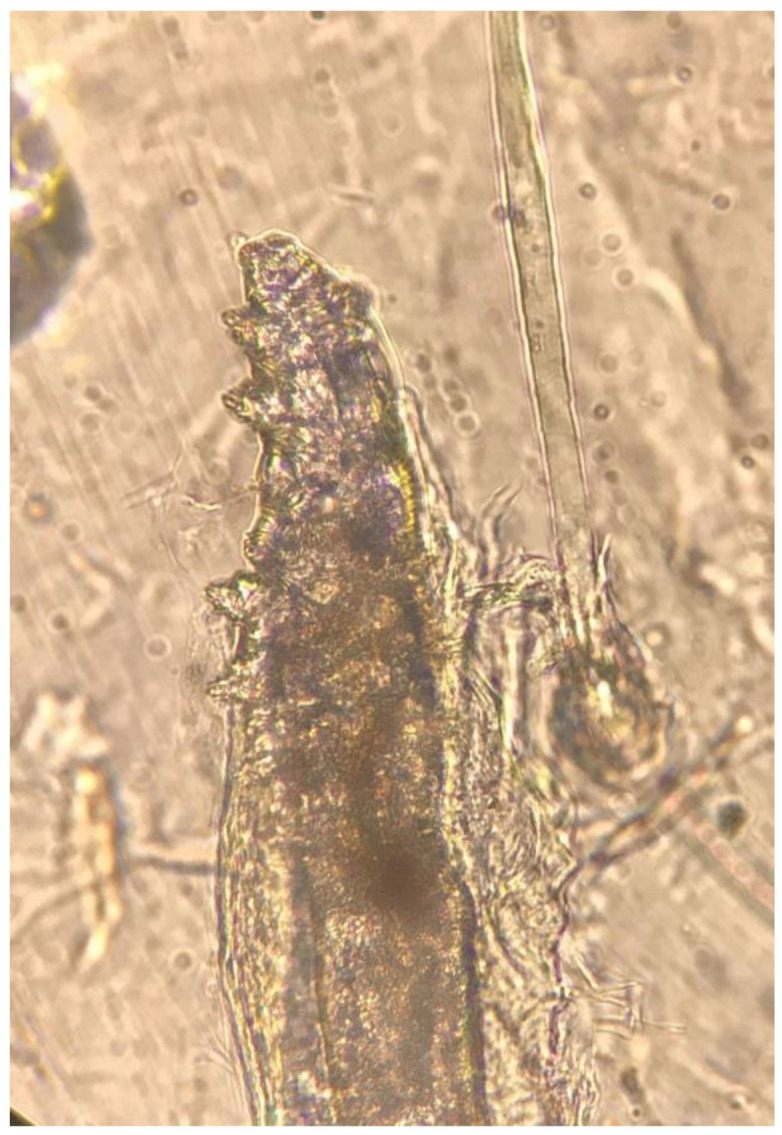
Single *Demodex* mite on standardized skin surface biopsies (SSSBs) under ×40 magnification by light microscope.

**Figure 2:**
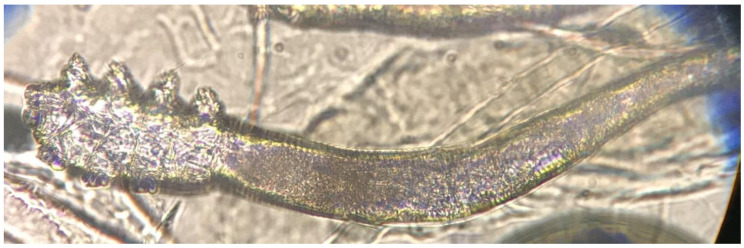
*Demodex* mite on SSSBs under ×40 magnification by light microscope.

The *Demodex* test was performed in both heart failure and age and sex-matched control groups. At least one *Demodex* mite was detected in 20 (56%) out of 36 HF patients and nine (25%) of control group (*p* = 0.008). (Figure 1 and Figure 2) The number of *Demodex* mites detected was significantly higher in the HF group (median 1; min. 0 and max. 10) compared to the control group (median 0; min. 0 and max. 3) (*p* < 0.001). Demodicidosis, which is defined as the determination of five or more *Demodex* mites in a 1-cm^2^ area, was positive in 14 (40%) HF patients. In none of the controls was demodicidosis detected (*p* < 0.001) (Table 2).

## 4. Discussion

In this study, increased *Demodex* density was observed in patients who were hospitalized for HF exacerbation compared to a healthy control group. Additionally, demodicidosis was significantly more common in HF patients. 

*Demodex* positivity was reported in asymptomatic healthy individuals but in low densities. Mother-to-infant transmission occurs soon after birth and their number increases by puberty. With aging, the percentage of individuals who are infected increases, reaching a peak in the fifth and sixth decades. The prevalence of *Demodex* infestation is nearly 95% in individuals higher than 71 years. Demodicidosis is considered when they multiply and reach to the ≥5 mites/cm^2^ of skin [14,15,22]. Although some local or systemic factors were suggested, the exact cause of this increase in density has not been clarified. Heart failure is usually seen in the elderly population. Although *Demodex* positivity is expected to be high in this age group, we found a significant increase in *Demodex* positivity and demodicidosis in HF patients compared to age and sex-matched healthy controls. 

Demodicidosis is common in immunocompromised patients. In acute lymphoblastic and myelocytic leukemia patients, demodicidosis was described and those taking cytosine arabinoside, daunorubicin, hydroxyurea and mitoxantrone treatments had the highest densities. [15,17,18,23,24]. Acquired immunodeficiency syndrome patients were also reported as having increased rates of demodicidosis. Therefore, the conditions and medications affecting humoral and cellular immunity might cause the proliferation of *Demodex* mites [17,25]. The activation or dysregulation of the immune system plays a major role in the development and progression of heart failure [26]. Therefore, the presence of demodicidosis in our heart failure patients could be related with this situation.

Patients with systemic diseases were also evaluated for *Demodex* positivity. End-stage-renal failure patients were found to have increased *Demodex* mites reaching the mean number of 6/cm^2^ [20,21]. In our study, the median number of *Demodex* mites was found to be 1; however, demodicidosis was detected in 39% of HF patients. Even though the median number is less compared to end-stage renal disease patients, the demodicidosis ratio is similar. These rates of *Demodex* positivity might support the potential shared mechanisms of immune system dysfunction in these patient groups.

Uremia in chronic renal failure patients was proposed to cause changes in immune response, such as impaired neutrophil and lymphocyte functions [27]. Thus, immune dysfunction allows for the growth of obligatory mites like *Demodex* species. Immune system activation and inflammation are considered to play a significant role in the progression of HF [26]. In particular, discrepancies in lymphocyte, monocyte, eosinophil and mast cells have been recognized in high-risk HF patients. Decreased lymphocyte count was found to be a poor prognostic factor in hospitalized chronic HF patients [28]. Okamato et al. demonstrated that circulating T regulatory cells were decreased in decompensated HF patients. This situation was associated with inflammation and left ventricular dysfunction. Therefore, T regulatory cells might play a critical role in controlling inflammation via the suppression of cellular immune responses. They also found that these T cells were an independent predictor of recurrent hospitalization in HF patients [29]. Although the infections were the cause of exacerbation in 19% of heart failure patients in our study, *Demodex* positivity and demodicidosis were detected in 56% and 39% of patients, respectively. A possible dysfunction in the immune system of HF patients might cause an increase in local infections of obligatory microorganisms like *Demodex* mites. 

The small number of patients and being a cross sectional study without long-term follow-up are limitations of this study. Prospective studies with larger patient populations would give more powerful data regarding the importance of *Demodex* positivity in HF patients. Additionally, studies with long-term follow-up enable us to get more data regarding the *Demodex* positivity in HF patients. 

In conclusion, this study shows that *Demodex* positivity and demodicidosis were common in hospitalized HF patients compared to healthy controls. Demodicidosis should be considered in hospitalized HF patients.

## Figures and Tables

**Table 1:** Baseline clinical and laboratory parameters of heart failure patients.

Clinical Characteristics	Heart Failure Group (*n =* 36)
Age (years) mean ± SD	67 ± 7
Female *n* (%)	11 (31%)
Male *n* (%)	25 (69%)
Ischemic etiology *n* (%)	17 (47%)
HFREF *n* (%)	19 (53%)
HFPEF *n* (%)	17 (47%)
AF *n* (%)	24 (67%)
LVEF (%) mean ± SD	40 ± 14
White blood cell count (×10^3^/uL) mean ± SD	7.2 ± 2.6
Hemoglobin (g/dL) mean ± SD	11.8 ± 1.5
Platelet (×10^3^/uL) mean ± SD	256 ± 105
Creatinine (mg/dL) mean ± SD	1.3 ± 0.4
eGFR (ml/min/1.73m^2^) mean ± SD	63 ± 31
AST (U/L) mean ± SD	23 ± 11
ALT (U/L) mean ± SD	18 ± 20
Oral anticoagulant use *n* (%)	23 (64%)
Reasons for exacerbation *n* (%)	
Fluid retention due to noncompliance	19 (53%)
Infections	7 (19%)
Arrhythmias	6 (17%)
Uncontrolled hypertension	2 (5.5%)
Others	2 (5.5%)

*n*: number, mean ± SD: mean ± standart deviation, HFREF: heart failure with reduced ejection fraction, HFPEF: heart failure with preserved ejection fraction, AF: atrial fibrillation, LVEF: left ventricular ejection fraction, eGFR: estimated glomerular filtration rate, AST: aspartate aminotransferase, ALT: alanine aminotransferase.

**Table 2:** Comparison of the patient and control groups.

	Patient Group (*n =* 36)	Control Group (*n =* 36)	*P* Value
Age (year) mean ± SD	67 ± 7	64 ± 6	0.068
Female *n* (%)	11 (31%)	14 (39%)	0.458
Male *n* (%)	25 (69%)	22 (61%)	0.458
*Demodex* positivity *n* (%)	20 (56%)	9 (25%)	**0.008**
Number of *Demodex* median (min, max)	1 (0, 10)	0 (0, 3)	**<0.001**
Demodicidosis *n* (%)	14 (40%)	0	**<0.001**

*n*: number, mean ± SD: mean ± standart deviation, min: minimum, max: maximum.
